# Sex differences in plasma p-tau181 associations with Alzheimer’s disease biomarkers, cognitive decline, and clinical progression

**DOI:** 10.1038/s41380-022-01675-8

**Published:** 2022-06-29

**Authors:** Amaryllis A. Tsiknia, Steven D. Edland, Erin E. Sundermann, Emilie T. Reas, James B. Brewer, Douglas Galasko, Sarah J. Banks, for the Alzheimer’s Disease Neuroimaging Initiative

**Affiliations:** 1 Department of Neurosciences, University of California, San Diego, La Jolla, CA 92093 USA; 2 Division of Biostatistics, School of Public Health and Human Longevity Science, University of California, San Diego, La Jolla, CA 92093 USA; 3 Department of Psychiatry, University of California, San Diego, La Jolla, CA 92093 USA; 4 Research Service, Veterans Affairs San Diego Healthcare System, San Diego, CA 92161 USA

**Keywords:** Neuroscience, Predictive markers

## Abstract

Studies have shown that women on the Alzheimer’s disease (AD) continuum have more pathological tau in the brain and cerebrospinal fluid (CSF), than men. Some studies have found that higher levels of tau biomarkers are more strongly associated with clinical AD, cognitive decline and neurodegeneration in women than in men. Despite major developments in the use of plasma tau phosphorylated at threonine 181 (p-tau181) as an AD biomarker, it is unknown whether these sex differences apply to plasma p-tau181. In 1060 Alzheimer’s Disease Neuroimaging Initiative (ADNI) participants (47% women, 73.8 ± 7.6 years old), we examined sex differences in plasma p-tau181 levels and their association with other biomarkers, cognitive decline and incident AD. Linear regressions tested for an effect of sex on plasma p-tau181 levels and for plasma p-tau181 × sex interactions on CSF p-tau181, as well as entorhinal cortex tau, cortical amyloid-β (Aβ) deposition, and brain glucose metabolism, quantified using PET imaging. Linear mixed effects models tested for a sex × baseline plasma p-tau181 interaction on change in cognition over time. Finally, Cox models tested for a sex × plasma p-tau181 interaction on the risk of AD dementia in participants who were free of dementia at baseline. Despite similar plasma p-tau181 levels between sexes, women had lower brain glucose metabolism, greater brain Aβ and entorhinal cortex tau deposition, higher CSF p-tau181 and faster cognitive decline in relation to higher baseline plasma p-tau181 levels compared with men. Among Aβ positive, dementia-free participants, women had higher rates of incident AD dementia associated with increasing baseline plasma p-tau181 levels, relative to men. Our results suggest that sex may impact the clinical interpretation of plasma p-tau181 concentrations. If replicated, these findings could have important implications for the use of plasma p-tau181 as an accessible AD biomarker and screening tool for preventive and therapeutic clinical trials.

## Introduction

Alzheimer’s disease (AD) presents a global health care challenge, affecting more than 1 in 9 adults over the age of 65 in the United States alone [1]. Although in-vivo measures of amyloid-β (Aβ) and tau pathology in the brain and cerebrospinal fluid (CSF) have substantially improved the accuracy of AD diagnosis and prognosis [2–4], they remain costly and invasive, making them largely inaccessible to primary clinical care settings [5]. Therefore, blood biomarkers of AD may be useful alternatives [5]. Recent studies showed that plasma tau phosphorylated at threonine 181 (p-tau181) can identify people with AD dementia, distinguish Aβ positive from Aβ negative individuals, and predict future dementia with a degree of accuracy that is comparable to the predictive accuracy of established AD biomarkers [6]. Furthermore, higher plasma p-tau181 at baseline is associated with higher levels of cortical tau measured by [^18^F] Flortaucipir PET (FTP-PET) imaging [7].

A growing body of evidence shows that women on the AD continuum have higher levels of tau in the CSF [8–10] and the brain [11, 12] during life and more tau tangles evident at autopsy [13, 14], compared to men. An autopsy study found that for every 1-unit increase in tau pathology, women exhibit a 13-fold increase in odds of clinical AD, compared with only a 1.4-fold increase in odds of clinical AD in men [14]. Another study showed that among individuals with higher levels of CSF tau, women display greater hippocampal atrophy and faster decline in executive function, compared to men [15]. Taken together with prior evidence of strong associations between tau deposition and clinical presentation of AD [16], it is likely that elevated quantity of, and vulnerability to, tau pathology in women, may contribute to their more precipitous progression of cognitive decline [17]. However, it is unknown whether similar sex differences exist in plasma p-tau181 and its association with cognitive decline, AD risk and other AD biomarkers. Examining potential sex differences in plasma p-tau181 could aid in the development of sex-specific guidelines for its use as a screening tool in clinical trials, as well as an accessible biomarker in primary care settings.

Therefore, we examined (a) whether cross-sectional and longitudinal levels of plasma p-tau181 in individuals across the cognitive and clinical continuum differ by sex, (b) whether sex modifies associations of plasma p-tau181 with CSF p-tau181, Aβ deposition, glucose metabolism and tau deposition in the brain, and (c) whether sex modifies the effect of baseline plasma p-tau181 on cognitive decline and progression to AD dementia. We hypothesized that women would display higher levels of plasma p-tau181 cross-sectionally. Furthermore, we expected that women would exhibit a faster increase in plasma p-tau181 levels longitudinally, compared to men. Additionally, we hypothesized that women would display stronger associations between plasma p-tau181 levels and other established AD biomarkers. Finally, we expected women to have faster rates of cognitive decline and greater risk of conversion to AD dementia in association with higher baseline plasma p-tau181 levels, compared with men.

## Materials and methods

### Study design

Data were obtained from the Alzheimer’s Disease Neuroimaging Initiative (ADNI) database [18]. The ADNI is an ongoing multi-site study using multi-modal neuroimaging, clinical and neuropsychological research methods to measure the progression of mild cognitive impairment (MCI) and early AD. The study was approved by the Institutional Review Board of each participating research site and all participants provided written consent. For more details regarding the ADNI procedures and diagnostic criteria, refer to www.adni-info.org. Briefly, participants with Mini Mental Status Examination (MMSE) scores between 24 and 30 (inclusive) and a Clinical Dementia Rating (CDR) of 0, who show no evidence of cognitive impairment or depression are classified as cognitively normal. Individuals who score between 24 and 30 (inclusive) on the MMSE, have a CDR of 0.5 and objective memory impairment as indicated by education-adjusted Wechsler Memory Scale Logical Memory II performance, and show no impairment in other domains of cognition and do not meet the diagnostic criteria for dementia, are diagnosed with MCI. Finally, participants with objective memory dysfunction, MMSE scores between 20 and 26 (inclusive), a CDR ≥ 0.5, who meet the NINDS/ADRDA criteria for probable AD are diagnosed with AD dementia. Data used for the present analysis were obtained from the Laboratory of Neuro Imaging (LONI) database (ida.loni.usc.edu) in June of 2021.

### Participants

We included all ADNI participants with at least one available plasma p-tau181 measurement and [^18^F] Florbetapir PET (FBP-PET) scan (for measurement of brain Aβ deposition and determination of Aβ positivity) at baseline (*n* = 1066). Six participants with plasma p-tau181 values greater than five standard deviations above the mean plasma p-tau181 concentration of the cohort were identified as outliers and were excluded from all subsequent analyses. A subset of participants had Fluorodeoxyglucose PET (FDG-PET) data (*n* = 1047), and CSF p-tau181 data (*n* = 859) acquired at the same visit as the plasma p-tau181 measurement and another subset had FTP-PET data (*n* = 304). Since no participants completed a FTP-PET scan at the same study visit at the plasma p-tau181 measurement, we selected the FTP-PET data obtained at the visit closest to the plasma p-tau181 measurement, which was obtained on average 5.24 years after the plasma p-tau181 measurement. Most participants also had longitudinal neuropsychological data available including Montreal Cognitive Assessment (MoCA) scores (*n* = 958), CDR– sum of boxes (CDR-SOB) scores (*n* = 829) and MMSE scores (*n* = 823). Annual follow-up plasma p-tau181 data was available for a subset of participants (*n* = 954). Subject exclusion and data availability for each subsample is detailed in Supplementary Fig. 1.

### Plasma p-tau181 measurement

ADNI procedures for blood sample collection, storage and processing have been described previously [19]. Plasma p-tau181 concentrations were determined using an in-house assay performed on a Simoa HD-X (Quanterix) at the Clinical Neurochemistry Laboratory, University of Gothenburg, Sweden, as previously described [20].

### Brain imaging acquisition and processing

Regional PET imaging data were obtained from the LONI database in June 2021 (ida.loni.usc.edu). ADNI brain imaging acquisition and processing procedures have been described in detail elsewhere http://adni.loni.usc.edu/methods/documents. Briefly, structural MRI scans are performed on 3 T scanners using either a 3D MPRAGE or IR-SPGR T1-weighted sequence with sagittal slices and spatial resolution of 1.1 × 1.1 × 1.2 mm^3^ and are then skull-stripped, segmented and parcellated using FreeSurfer (version 5.3.0). FBP-PET images are acquired 50–70 min post-injection in a series of four 5-minute frames while FTP-PET and FDG-PET images are acquired 75–105 min and 30–60 min post-injection, respectively, in a series of six 5-min frames. Each frame is coregistered to the first image to account for participant motion. The motion-corrected dynamic image set is then averaged and smoothed to a uniform isotropic resolution of 8 mm full width at half maximum and coregistered with the participant’s structural scan. Standard uptake value ratios (SUVRs) of FTP uptake are computed for each FreeSurfer-derived region by referencing to mean cerebellar gray matter retention, and corrected for partial volume effects using the Geometric Transfer Matrix approach [21–25]. We used a bilateral volume-weighted entorhinal cortex (EC) SUVR, given that it is a cortical site of early AD-related tau deposition [26–28]. To determine Aβ deposition we used a cortical summary SUVR, which is intensity normalized by mean whole-cerebellum retention. To determine Aβ positivity we compared this cortical summary value to a SUVR cut-off value of 1.11 [27, 28]. Finally, to determine brain glucose metabolism, we used the FDG-PET SUVR of a volume-weighted average of a pons/vermis intensity-normalized region including the bilateral angular and inferior temporal gyrus, and posterior cingulate, which have been shown to be particularly vulnerable to AD-related changes in glucose metabolism [29].

### Statistical analyses

All statistical analyses were performed using RStudio (version 1.4.1106).

#### Sex differences in demographic and clinical characteristics

Sex differences in demographic variables were assessed using an independent samples t-test for continuous variables and a chi-squared test for categorical variables (Table 1).

**Table 1:** Demographic and clinical sample characteristics at baseline shown by sex.

Characteristic		Men (*n* = 566)	Women (*n* = 494)	Total (*N* = 1060)	*P* value
Age at baseline, years (SD)		74.8 (7.4)	72.8 (7.6)	73.8 (7.6)	<0.001
*APOE* ε4 carriers, No. (%)		249 (44.0)	213 (43.1)	462 (43.6)	0.778
Diagnosis	Cognitively normal, No. (%)	168 (29.6)	191 (38.6)	359 (33.9)	0.008
	Mild cognitive impairment, No. (%)	290 (51.2)	226 (45.7)	516 (48.7)	
	Dementia, No. (%)	108 (19.0)	77 (15.6)	185 (17.5)	
Aβ positive, No. (%)		292 (51.2)	268 (54.3)	560 (52.8)	0.385
Education, years (SD)		16.7 (2.6)	15.7 (2.7)	16.2 (2.7)	<0.001
Race	White, No. (%)	532 (94.0)	450 (91.1)	982 (92.6)	0.027
	Black or African American, No. (%)	14 (2.5)	27 (5.5)	41 (3.9)	
	Asian, No. (%)	12 (2.1)	6 (1.2)	18 (1.7)	
	Native Hawaiian or Other Pacific Islander, No. (%)	0	2 (0.4)	2 (0.2)	
	American Indian or Alaskan Native, No. (%)	1 (0.2)	0	1 (0.1)	
	More than one race or Unknown, No. (%)	7 (1.2)	9 (1.8)	16	
Body Mass Index, kg/m^2^ (SD)		27.3 (4.2)	27.2 (6.1)	27.3 (5.2)	0.8
Plasma p-tau181, pg/mL (SD)		19.0 (10.8)	17.1 (10.1)	18.1 (10.5)	0.003
Global Aβ PET, SUVR (SD)		1.20 (0.2)	1.22 (0.2)	1.21	0.21
CSF p-tau181, pg/mL (SD)		25.7 (12.3)	28.1 (16.0)	26.8 (14.2)	0.014
Composite Fluorodeoxyglucose-PET, SUVR (SD)		1.25 (0.2)	1.28 (0.2)	1.26 (0.2)	0.001
Entorhinal cortex tau PET, SUVR (SD)		1.9 (0.7)	2.1 (0.9)	2.0 (0.8)	0.05

#### Sex differences in plasma p-tau181

To test the hypothesis that women had higher plasma p-tau181 levels at baseline, relative to men, we used a linear regression model testing for an effect of sex on plasma p-tau181 levels at baseline with covariates for *APOE* ε4 status, age and years of education. To determine whether women exhibited a faster increase in plasma p-tau181 concentration over time, compared to men, we used a linear mixed effects (LME) model with random slopes and intercepts. The LME model tested for an interaction between sex and time on longitudinal changes in plasma p-tau181 after adjusting for age, *APOE* ε4 status and education. Given prior evidence that sex differences in CSF p-tau181 are modulated by *APOE* ε4 status [8–10], we conducted an exploratory analysis of an interaction between sex and *APOE* ε4 status on plasma p-tau181 levels cross-sectionally and longitudinally, while adjusting for age and years of education.

#### Sex differences in associations between plasma p-tau181 and AD biomarkers

To examine whether sex modifies cross-sectional associations between plasma p-tau181 and FBP-PET, FDG-PET, FTP-PET and CSF p-tau181 we used age-, *APOE* ε4 status-, and education-adjusted linear regression models testing for an interaction between sex (coded as 1 = female, 0 = male) and plasma p-tau181 on each of the aforementioned biomarkers. Due to the substantial time difference between the plasma p-tau181 measurement and FTP-PET scan, for models of FTP-PET we also included the time-lag between the plasma measurement and FTP-PET scan as a covariate.

#### Sex differences in the association between baseline plasma p-tau181 and cognitive decline

To examine whether sex modifies the effect of baseline plasma p-tau181 levels on longitudinal MMSE, CDR-SOB and MoCA scores we used LMEs with random intercepts and slopes. We covaried for age, education, and *APOE* ε4 status. Given the potential clinical applicability of plasma p-tau181 cut-off points, we ran exploratory analyses of sex differences in associations between plasma p-tau181 and cognitive decline with models treating baseline plasma p-tau181 as a dichotomous variable, using an ADNI-based cut-off value of >21.99 pg/mL [7]. All analyses were performed on the entire cohort, as well as groups stratified by diagnosis and Aβ status.

#### Sex differences in plasma p-tau181 associated risk of dementia

For this analysis, we used Cox proportional hazard models to examine whether sex modifies the association between baseline plasma p-tau181 and rate of incident dementia in 843 dementia-free participants who had follow-up diagnostic data for up to 9 years. Participants were censored at their last available follow-up diagnostic examination. We used Martingale and Schoenfeld residuals to test for the assumption of linearity and proportionality of hazards, respectively.

A detailed summary of our aims, hypotheses and associated statistical tests can be found in Supplementary Table 1. Standardized beta coefficients, standard errors and p values are reported for linear regression and linear mixed effects models, while hazard ratios (HR), 95% confidence intervals and p values are reported for the Cox proportional hazards model. *P* values smaller than 0.05 were considered significant. In the case of a significant sex interaction in linear regression, LME and Cox proportional hazard models, we additionally reported effect sizes for men and women separately, which we derived from the interaction model using the *multcomp* R package.

## Results

### Sex differences in demographic and clinical characteristics

In our sample of 1060 participants, there were 494 (46.7%) women and 566 (53.4%) men. Across all participants, 359 (33.9%) were cognitively normal (CN), 516 (48.7%) were diagnosed with MCI, 185 (17.5%) were diagnosed with dementia, and 560 (52.8%) were Aβ positive. Our sample was predominantly White (92.6%) with a mean age of 73.8 and an average of 16.2 years of education. Women in our sample were younger, had fewer years of education, higher levels of CSF p-tau181 and brain glucose metabolism and were more likely to be CN and less likely to have MCI or dementia, compared to men (Table 1). To account for sex differences in diagnostic status we additionally adjusted for the effect of diagnosis in subsequent analyses.

### Sex differences in plasma p-tau181

We observed no significant sex differences in baseline plasma p-tau181 levels among the whole cohort and any of the stratified groups. There were no sex differences in the rates of plasma p-tau181 change over time in the overall cohort and the stratified groups. Our exploratory analysis of a potentially modulating effect of *APOE* ε4 status on sex differences in plasma p-tau181 revealed no significant sex × *APOE* ε4 status interaction on plasma p-tau181 levels cross-sectionally or longitudinally.

### Sex differences in associations between plasma p-tau181 and AD biomarkers

**Fig. 1:**
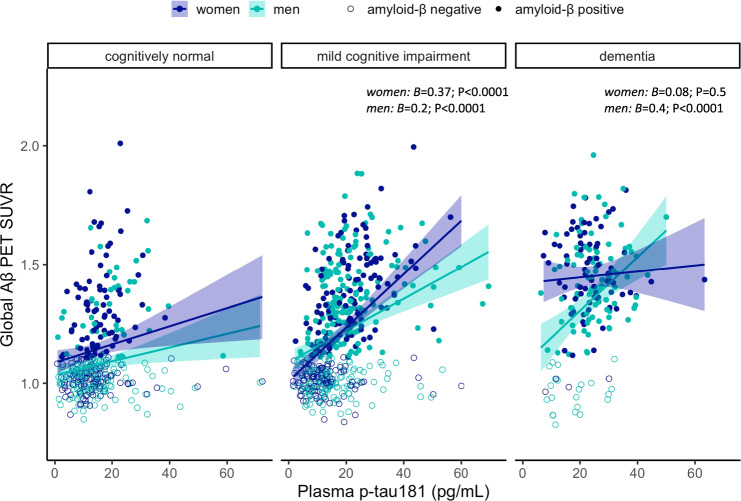
Sex modulates plasma p-tau181 associations with brain amyloid-β deposition. Scatter plots demonstrating sex differences in the cross-sectional association between plasma p-tau181 levels and brain amyloid-β deposition (measured by amyloid-β PET in cortical summary region) in each diagnostic group. SUVR standardized uptake value ratio.

We observed no significant interaction between sex and plasma p-tau181 on FBP-PET SUVR in the whole cohort. In diagnosis-stratified analyses (Fig. 1), there was a significant interaction between sex and plasma p-tau181 on FBP-PET SUVR in the MCI group (*B* = 0.17; SE = 0.07; *P* = 0.017), whereby the association between higher plasma p-tau181 and higher FBP-PET SUVR was stronger for women with MCI (*B* = 0.37; SE = 0.06; *P* < 0.0001), compared to men with MCI (*B* = 0.2; SE = 0.05; *P* < 0.0001). However, among individuals with dementia, we found an interaction between sex and plasma p-tau181 on FBP-PET SUVR (*B* = −0.3; SE = 0.16; *P* = 0.03), such that higher plasma p-tau181 was associated with higher FBP-PET SUVR among men (*B* = 0.4; SE = 0.1; *P* < 0.0001), but not women (*B* = 0.08; SE = 0.1; *P* = 0.5). There was no interaction between sex and plasma p-tau181 levels on FBP-PET SUVR in CN participants or in Aβ stratified groups.

**Fig. 2:**
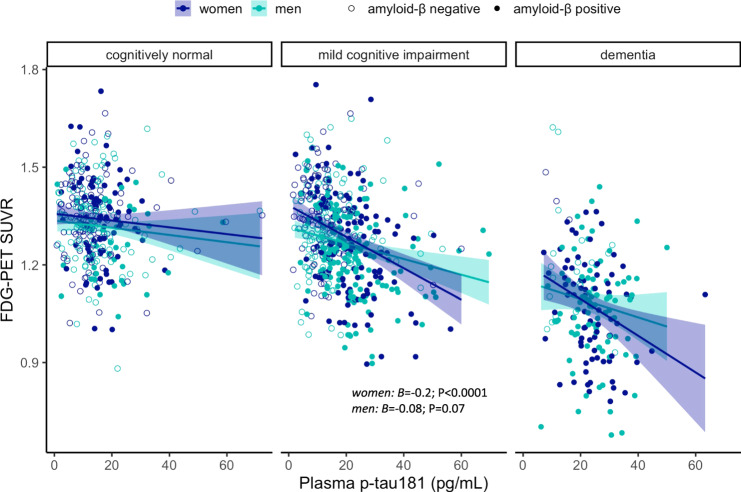
Sex modulates plasma p-tau181 associations with cerebral glucose metabolism. Scatter plots depicting sex differences in the cross-sectional association between plasma p-tau181 levels and brain glucose metabolism (measured by FDG-PET) in each diagnostic group. SUVR standardized uptake value ratio.

We found a significant interaction between sex and plasma p-tau181 on FDG-PET SUVR in the whole sample (*B* = −0.1; SE = 0.05; *P* = 0.026), whereby women displayed lower brain glucose metabolism with increasing plasma p-tau181 levels (*B* = −0.2; SE = 0.04; *P* < 0.0001), than did men (*B* = −0.09; SE = 0.04; *P* = 0.01). In stratified analyses (Fig. 2), the interaction was significant within Aβ positive individuals (*B* = −0.23; SE = 0.08; *P* = 0.003) and participants with MCI (*B* = −0.14; SE = 0.7; *P* = 0.04), but not within Aβ negative participants or the other diagnostic groups. The association between plasma p-tau181 and FDG-PET was stronger in Aβ positive women (*B* = −0.3; SE = 0.06; *P* < 0.0001) than Aβ positive men (*B* = −0.1; SE = 0.05; *P* = 0.04), but it was statistically significant within both sexes. Among individuals with MCI, higher plasma p-tau181 was associated with lower FDG-PET SUVR among women (*B* = −0.2; SE = 0.05; *P* < 0.0001) but not men (*B* = −0.08; SE = 0.05; *P* = 0.07).

**Fig. 3:**
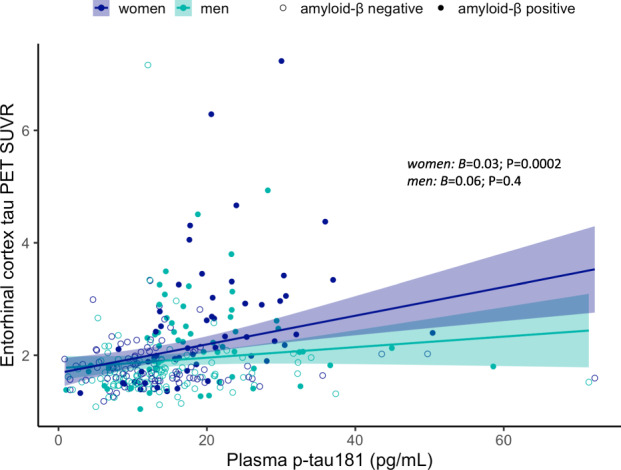
Sex modulates the association between plasma p-tau181 and entorhinal cortex tau deposition. Scatter plot depicting sex differences in the cross sectional association between plasma p-tau181 levels and entorhinal cortex tau deposition (measured using tau PET). SUVR standardized uptake value ratio.

We also observed an interaction between sex and plasma p-tau181 on EC FTP-PET in the whole sample (Fig. 3, *B* = 0.2; SE = 0.1; *P* = 0.03), whereby higher plasma p-tau181 correlated with higher EC FTP-PET SUVR among women (*B* = 0.03; SE = 0.08; *P* = 0.0002), but not men (*B* = 0.06; SE = 0.07; *P* = 0.4). This interaction was not significant in diagnosis- and Aβ-stratified groups, likely due to the smaller sample sizes.

**Fig. 4:**
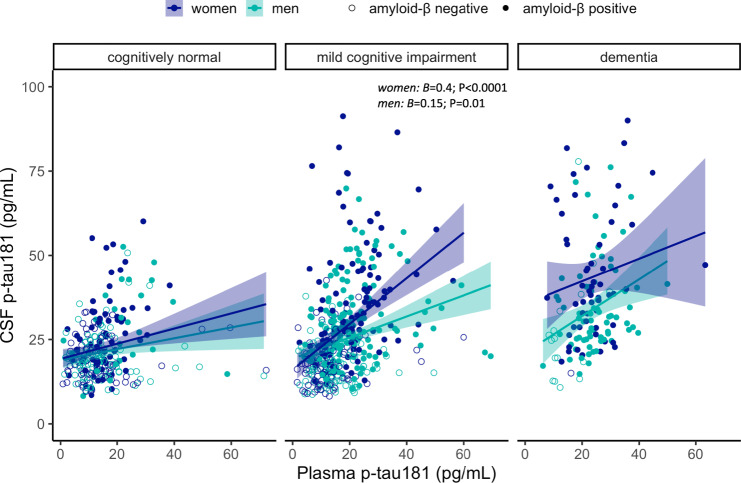
Sex modulates plasma p-tau181 associations with CSF p-tau181. Scatter plots depicting sex differences in the cross-sectional association between plasma p-tau181 and CSF p-tau181 in each diagnostic group.

Finally, we found a significant interaction between sex and plasma p-tau181 on CSF p-tau181 levels in the entire cohort (*B* = 0.2; SE = 0.06; *P* = 0.002). Though the association between higher plasma p-tau181 and higher CSF p-tau181 was stronger for women (*B* = 0.3; SE = 0.04; *P* < 0.00001) than men (*B* = 0.1; SE = 0.04; *P* = 0.001), it was statistically significant for both sexes. In stratified analyses (Fig. 4), the sex × plasma p-tau181 interaction was significant in the Aβ positive (*B* = 0.2; SE = 0.1; *P* = 0.02) and MCI group (*B* = 0.2; SE = 0.08; *P* = 0.003), but not within Aβ negative participants and other diagnostic groups. Within the Aβ positive and MCI groups, the correlation between higher plasma p-tau181 and higher CSF p-tau181 was significant in both sexes. However, this association was stronger for Aβ positive and MCI women (*B* = 0.4; SE = 0.08; *P* < 0.0001 and *B* = 0.4; SE = 0.07; *P* < 0.0001, respectively) than it was for Aβ positive and MCI men (*B* = 0.15; SE = 0.06; *P* = 0.02 and *B* = 0.15; SE = 0.06; *P* = 0.01 respectively).

### Sex differences in the association between baseline plasma p-tau181 and cognitive decline

**Fig. 5:**
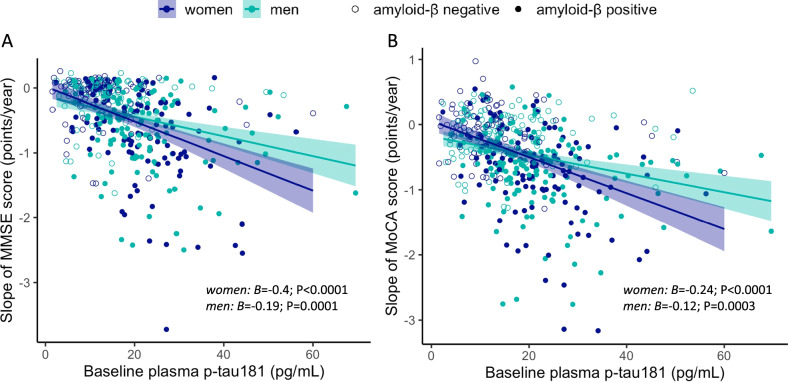
Sex modulates the association between baseline plasma p-tau181 levels and cognitive decline. Scatter plots demonstrating sex differences in the association between baseline plasma p-tau181 levels and decline in **A** MMSE and **B** MoCA performance over time in participants diagnosed with mild cognitive impairment. MMSE Mini Mental Status Examination, MoCA Montreal Cognitive Assessment.

There was a significant interaction between sex and baseline plasma p-tau181 levels on MMSE scores over time within the whole cohort, such that women displayed faster decline in MMSE scores in relation to elevated plasma p-tau181 levels at baseline (*B* = −0.01; SE = 0.05; *P* = 0.04). Higher baseline plasma p-tau181 correlated with faster decline in MMSE performance for both sexes but the association was stronger for women (*B* = −0.25; SE = 0.03; *P* < 0.0001), than for men (*B* = −0.16; SE = 0.03; *P* < 0.0001). The sex × plasma p-tau181 interaction on MMSE scores over time was significant in the Aβ positive (*B* = −0.21; SE = 0.09; *P* = 0.03) and MCI group (*B* = −0.19; SE = 0.07; *P* = 0.009). Within Aβ positive individuals and participants with MCI (Fig. 5A), higher baseline plasma p-tau181 levels predicted faster decline in MMSE scores in both sexes, but this effect was stronger for women in the Aβ positive and MCI groups (*B* = −0.45; SE = 0.07; *P* < 0.0001 and *B* = −0.4; SE = 0.05; *P* < 0.0001, respectively), than men (*B* = −0.25; SE = 0.06; *P* = 0.0001 and *B* = −0.19; SE = 0.08; *P* = 0.0001, respectively).

Women in our sample displayed faster decline in MoCA scores in association with higher baseline plasma p-tau181 levels compared to men (*B* = −0.08; SE = 0.03; *P* = 0.019). Though the association between higher plasma p-tau181 and faster decline in MoCA scores was significant in both sexes, it was stronger for women (*B* = −0.19; SE = 0.02; *P* < 0.0001), than men (*B* = −0.11; SE = 0.03; *P* < 0.0001). In stratified analyses, this sex difference was significant in the MCI group (*B* = −0.12; SE = 0.05; *P* = 0.013) but not in the Aβ positive, Aβ negative and other diagnostic groups. Among individuals with MCI (Fig. 5B), higher baseline plasma p-tau181 was associated with faster decline in MoCA scores for both sexes, but the effect was stronger for women (*B* = −0.24; SE = 0.037; *P* < 0.0001), than men (*B* = −0.12; SE = 0.03; *P* = 0.0003). We observed no significant interaction between sex and baseline plasma p-tau181 levels on change in CDR-SOB scores. Models treating baseline plasma p-tau181 as a dichotomous variable yielded similar results (Supplementary Fig. 1).

### Sex differences in the plasma p-tau181 associated risk of dementia

We found no sex differences in the association between plasma p-tau181 and the risk of incident dementia in the total sample of participants who were free of dementia at baseline. However, in stratified analyses, we found that sex modified the plasma p-tau181 associated risk of dementia in the Aβ positive group (*P* = 0.03). Specifically, each unit increase in baseline plasma p-tau181 levels was associated with a 7% increase in the rate of converting to dementia for Aβ positive women (hazard ratio (HR) = 1.07; 95% CI, 1.04–1.09; *P* = 4.8 × 10^−8^) and a 3% increase in the rate of converting to dementia for Aβ positive men (HR = 1.03; 95% CI, 1.01–1.04; *P* = 0.0003) after accounting for age. The interaction between sex and plasma p-tau181 levels on the rate of dementia was not significant in the Aβ negative, CN and MCI groups.

## Discussion

In this ADNI sample of clinically normal older adults and individuals on the AD continuum, we found that although plasma p-tau181 concentrations were similar between sexes, women had worse phenotypic biomarker profiles in association with elevated plasma p-tau181 concentrations, than men. Specifically, high plasma p-tau181 levels were more strongly associated with greater cortical Aβ deposition, higher concentrations of p-tau181 in the CSF, greater future EC tau deposition and lower brain glucose metabolism among women, compared with men. Furthermore, we observed faster cognitive decline and greater risk of conversion to dementia in association with higher baseline plasma p-tau181 levels among women, compared with men. Notably, these sex differences were driven by Aβ positive participants and individuals with MCI.

If replicated in larger, population-based samples, our findings could have important implications for the use plasma p-tau181 as a screening tool for clinical trials and as an accessible biomarker of AD. Moscoso et al. recently demonstrated that plasma p-tau181 can help identify target populations for enrollment in preventive and therapeutic clinical trials whilst dramatically reducing biomarker-screening costs [30]. However, its use in clinical trial recruitment would require the development of cutoffs that distinguish high-risk from low-risk individuals with maximal sensitivity. For a given plasma p-tau181 concentration, women in our study exhibited worse biomarker profiles and greater risk of cognitive decline and AD dementia, compared to men. Therefore, the use of a single plasma p-tau181 cutoff for clinical trial enrollment may result in the inclusion of women with more progressive AD neuropathology who may be less likely to benefit from preventive interventions. A recent review of sex differences in the measurement and interpretation of fluid biomarkers of AD [31] suggested that the presence of sex differences in the clinical susceptibility associated with an AD biomarker may warrant the development of sex-specific cut-points even in the absence of sex differences in the concentration of that biomarker. Future investigation is warranted to determine whether the use of sex-specific plasma p-tau181 cutoffs can enhance the sensitivity of plasma p-tau181 as prognostic AD biomarker and a clinical trial screening tool.

We found no evidence to support the hypothesis that women have higher levels of plasma p-tau181 than men. Our supplemental analysis exploring a potential modulating effect of *APOE* ε4 status on sex differences in plasma p-tau181 levels revealed no significant sex × *APOE* ε4 status interaction on plasma p-tau181 concentration. This finding is at odds with several reports of higher CSF p-tau181 levels in women compared to men, particularly among *APOE* ε4 carriers [8–10]. Although plasma p-tau181 is assumed to be exclusively derived from the CSF [32], tau clearance mechanisms remain elusive [33]. A comprehensive review of potential CSF clearance pathways of AD pathology suggests that tau most likely crosses the blood-brain barrier (BBB) under conditions in which BBB permeability is increased [33]. Studies report higher levels of BBB permeability among men under both physiological and pathological conditions, compared to women of similar age [34, 35]. Furthermore, studies of induced pluripotent stem cells (iPSC) demonstrate lower permeability in female iPSC-derived brain microvascular endothelial cells, which are major cellular components of the BBB regulating the transfer of solutes, compared to male iPSC-derived endothelial cells [36]. A potentially “leakier” male BBB may contribute to similar plasma p-tau181 concentrations among men and women, despite higher CSF p-tau181 among women. Conversely, higher levels of tau pathology in the brain can trigger a cascade of neuroinflammatory responses leading to BBB breakdown [37]. Studies suggest that aquaporin 4, a water channel that is critically involved in the glymphatic clearance of CSF solutes, is downregulated in AD, which may lead to reduced tau clearance from the central nervous system [38–40]. Future studies should address the extent to which sex differences in tau clearance pathways and tau-mediated BBB breakdown exist and determine whether they impact sex differences in plasma versus CSF p-tau181 concentrations.

Prior studies have shown that plasma p-tau181 levels are associated with markers of Aβ pathology particularly in preclinical and prodromal AD, and demonstrate that these associations become weaker at advanced clinical stages [20, 41, 42]. Our study extends prior findings to report that the relative strength of the association between plasma p-tau181 and Aβ deposition at different disease stages, varies by sex. Among participants with MCI, women exhibited stronger associations between plasma p-tau181 and Aβ deposition than men, whereas in individuals with dementia, the association between plasma p-tau181 and Aβ deposition was observed only among men. Studies have shown that during the MCI stage, women outperform men in verbal memory tasks despite similar levels of hippocampal atrophy, brain hypometabolism and Aβ deposition [43–45]. Given that the diagnosis of MCI and AD in ADNI relies heavily on verbal memory test performance, this female verbal memory advantage may lead to more women with advanced AD neuropathology to be given a diagnosis of MCI instead of AD. Considering prior evidence that Aβ accumulation plateaus at more advanced neuropathological and clinical stages of AD [46], a delay in AD diagnosis among women in our sample might lead to greater Aβ saturation effects among women in the dementia group. This may explain the lack of an association between plasma p-tau181 and Aβ deposition in women with dementia in our study. Future investigation of sex differences in the association between plasma p-tau181 and Aβ deposition within diagnostic groups determined using additional memory tests or sex-specific verbal memory norms are necessary to assess this possible interpretation of our findings.

Furthermore, associations of plasma p-tau181 levels with brain glucose metabolism and EC tau deposition were modified by sex, whereby for a given plasma p-tau181 concentration, women displayed lower brain glucose metabolism and greater EC tau deposition than men. Interestingly, among participants with MCI, higher plasma p-tau181 levels were associated with lower brain glucose metabolism in women but not men. These results suggest that subtle increases in plasma p-tau181 levels at the MCI stage may be indicative of progressive neurodegeneration occurring among women, but not men. Thus, sex and diagnostic status should be critically considered in future assessments of plasma p-tau181 performance as a biomarker of progressive AD-related neurodegeneration and tau accumulation.

Sex modified the association between baseline plasma p-tau181 levels and cognitive decline, such that higher baseline plasma p-tau181 levels were predictive of faster decline in MoCA and MMSE, and CDR-SOB performance among women, compared with men. This sex difference was driven by individuals who were Aβ positive and participants with MCI. Furthermore, we found that Aβ positive women had a higher rate of incident dementia in relation to higher baseline plasma p-tau181, compared to Aβ positive men. Our findings are consistent with studies demonstrating that in the presence of elevated tau burden in the brain and CSF, women display faster cognitive decline trajectories [15], and higher rates of clinical AD [14], compared to men. Studies have shown that although women display greater cognitive resilience during early pathological AD stages, they decline faster clinically at more advanced pathological stages [43–45, 47–50]. Therefore, it is likely that some women in the Aβ positive and MCI group in our study are passed the pivotal point on their AD trajectory beyond which their clinical progression begins to accelerate. This interpretation of our findings is consistent with the results of our cross-sectional analysis, demonstrating that among participants with MCI, higher plasma p-tau181 levels are associated with lower brain glucose metabolism and EC tau deposition only among women. Given that imaging markers of brain hypometabolism and tau deposition are closely linked with subsequent cognitive impairment, the steeper decline in cognitive performance among women with higher levels of plasma p-tau181 may be partly due to the presence of more established levels of tau deposition and neurodegeneration.

### Limitations

Our study has some limitations. Our sample consisted of predominantly non-Hispanic White participants, which limits the generalizability of our findings. One study showed that sex differences in clinical AD trajectories vary between ethnic and racial groups [51]. Future studies should examine whether sex impacts the clinical interpretation of plasma p-tau181 concentrations in diverse, population-based samples. Additionally, our measure of EC tau deposition was obtained from PET scans acquired several years after plasma p-tau181 was measured. Although we adjusted our models for this time-lag, we were unable to draw conclusions regarding the temporality of the relationship between plasma p-tau181 levels and cortical tau deposition.

## Conclusions

In conclusion, our findings demonstrate that although women have similar levels of plasma p-tau181 with men, they display worse phenotypic biomarker profiles, faster rates of cognitive decline and higher odds of AD dementia in relation to elevated plasma p-tau181 concentrations, compared with men. Additionally, our study shows that plasma p-tau181 levels may be reflective of different neuropathological processes in men and women at a given stage of the AD continuum. Ultimately, our findings suggest that sex may impact the clinical interpretation of plasma p-tau181 concentrations and should be considered as a potential modifier of the prognostic utility of plasma p-tau181 in future studies. Further investigation of sex differences will be critical to the optimization of precision-medicine guidelines regarding the use of plasma p-tau181 as a non-invasive, and accessible AD biomarker.

## Supplementary information

Supplemental Material

## Data Availability

Data used in preparation of this article were obtained from the Alzheimer’s Disease Neuroimaging Initiative (ADNI) database (adni.loni.usc.edu). As such, the investigators within the ADNI contributed to the design and implementation of ADNI and/or provided data but did not participate in analysis or writing of this report.
