# Supplementary material for: Sex differences in plasma p-tau181 associations with Alzheimer’s disease biomarkers, cognitive decline, and clinical progression
Source: Mol Psychiatry. 2022 Jun 29;27(10):4314–22. doi: 10.1038/s41380-022-01675-8 (PMC9718670; doi:10.1038/s41380-022-01675-8)
Supplement: Supplementary file 1 — Supplemental Material [file 41380_2022_1675_MOESM1_ESM.pdf]

SUPPLEMENTARY INFORMATION: Sex differences in plasma p-tau181 associations with Alzheimer's disease biomarkers, cognitive decline, and clinical progression

Amaryllis A. Tsiknia[1], MS, Steven D. Edland[1,2], PhD, Erin E. Sundermann[3,4], PhD, Emilie T. Reas[1], PhD, James B. Brewer[1], MD, PhD, Douglas Galasko[1], MD, Sarah J. Banks*[1,3], PhD for the Alzheimer's Disease Neuroimaging Initiative**

[1]Department of Neurosciences, University of California, San Diego, La Jolla, CA 92093, USA

[2]Division of Biostatistics, School of Public Health and Human Longevity Science, University of California, San Diego, La Jolla, CA 92093, USA

[3]Department of Psychiatry, University of California, San Diego, La Jolla, CA 92093, USA

[4]Research Service, Veterans Affairs San Diego Healthcare System, San Diego, CA 92161, USA

*Address Correspondence to Dr. Sarah J. Banks, PhD, University of California, San Diego, School of Medicine, Department of Neurosciences, 9500 Gilman Drive, La Jolla, CA 92093, USA, sbanks@health.ucsd.edu, 858-246-1264

Supplementary Figure 1. Flow diagram of participant inclusion and arrival at the final study sample for each analysis.

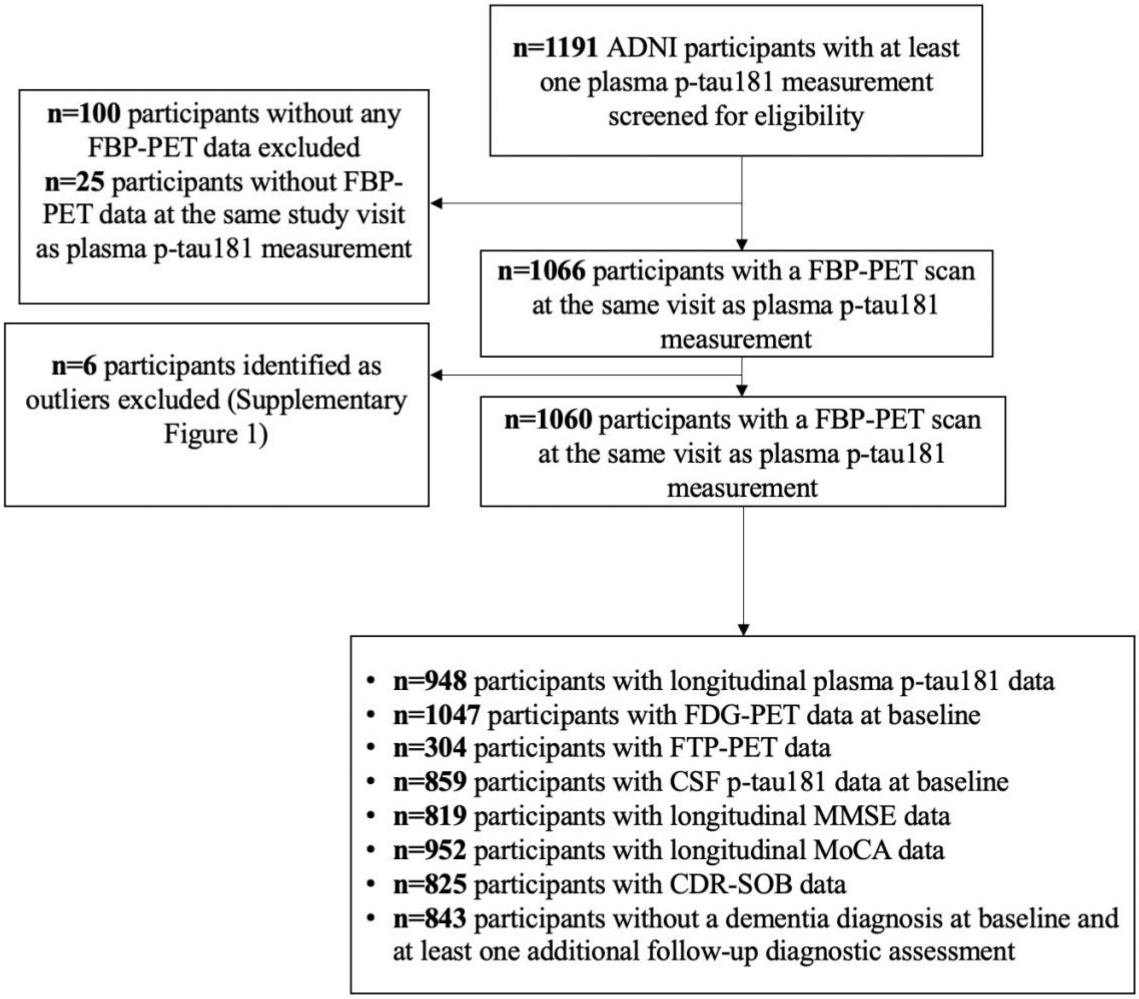

Supplementary Table 1. Summary of the study's main aims, hypotheses and statistical models used.

Aim	Hypothesis	Statistical Model
Aim 1: Examine sex differences in plasma p-tau181 levels cross-sectionally	We expected women to have higher levels of plasma p-tau181 than men at baseline.	linear regression model<br>[plasma p-tau181 ~ sex + age + *APOE* ε4 + education]
Aim 2: Examine sex differences in plasma p-tau181 longitudinally	We expected women accumulate plasma p-tau181 at a faster rate longitudinally	linear mixed effects model with random intercept and slope<br>[plasma p-tau181 ~ time × sex + age + *APOE* ε4 + education]
Aim 3: Determine whether sex modifies cross-sectional associations of plasma p-tau181 with Aβ deposition, glucose metabolism and tau deposition in the brain, and CSF p-tau181.	we expected sex to modify associations between plasma p-tau181 and AD biomarkers. Specifically, we hypothesized that higher plasma p-tau181 levels would be associated with worse biomarker profiles more strongly among women, relative to men.	linear regression models<br>• [FBP-PET ~ sex × plasma p-tau181 + age + *APOE* ε4 + education]<br>• [FDG-PET ~ sex × plasma p-tau181 + age + *APOE* ε4 + education]<br>• [FTP-PET ~ sex × plasma p-tau181 + age + *APOE* ε4 + education + time-lag]<br>• [CSF p-tau181 ~ sex × plasma p-tau181 + age + *APOE* ε4 + education]
Aim 4: Determine whether sex modifies the association between baseline plasma p-tau181 and longitudinal changes in cognitive performance	We expected women to exhibit faster cognitive decline in relation to higher baseline plasma p-tau181 levels, compared with men.	linear mixed effects model with random intercept and slope<br>• [MMSE ~ plasma p-tau181 × time × sex + age + *APOE* ε4 + education]<br>• [MoCA ~ plasma p-tau181 × time × sex + age + *APOE* ε4 + education]<br>• [CDR-SOB ~ plasma p-tau181 × time × sex + age + *APOE* ε4 + education]
Aim 5: Examine sex differences in the association between baseline plasma p-tau181 levels and dementia risk.	We hypothesized that higher baseline plasma p-tau181 levels would be associated with a higher risk of AD dementia among women, compared to men.	Cox proportional hazards model<br>[time, dementia diagnosis ~ plasma p-tau181 × sex + age]

Supplementary Figure 2. Average cognitive performance over time on the (A) MMSE and (B) MoCA among MCI participants, and the (C) CDR-SOB in amyloid-β positive participants shown by sex and baseline plasma p-tau181 status.

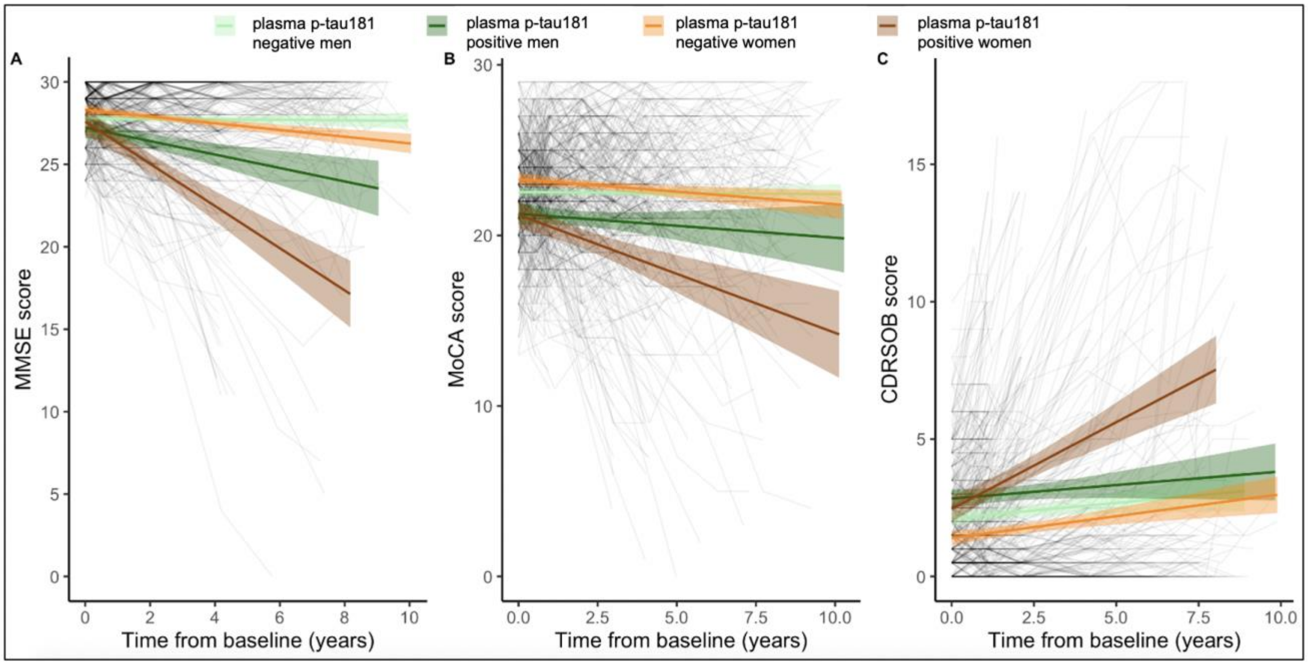

Among MCI patients, abnormal levels of baseline plasma p-tau181 were associated with faster MMSE score decline (A) among women with MCI (*B*=-0.85; SE=0.12; P<0.0001), than men with MCI (B=-0.45; SE=0.11; P<0.0001). Similarly, women with MCI displayed faster decline in MoCA performance (B) in association with abnormal plasma p-tau181 levels (*B*=-0.6; SE=0.08; P<0.0001), compared to men with MCI (*B*=-0.3; SE=0.08; P=0.0003). Finally, abnormal baseline plasma p-tau181 status was associated with faster increase in CDR-SOB scores (C) for Aβ positive women (*B*=0.67; SE=0.14; P<0.0001), but not Aβ positive men (*B*=0.21; SE = 0.13; P=0.1).
